# Assessing the Interfacial Dynamic Modulus of Biological Composites

**DOI:** 10.3390/ma14123428

**Published:** 2021-06-21

**Authors:** Yaniv Shelef, Avihai Yosef Uzan, Ofer Braunshtein, Benny Bar-On

**Affiliations:** 1 Department of Mechanical Engineering, Ben-Gurion University of the Negev, Beer Sheva 84105, Israel; shelefy@post.bgu.ac.il (Y.S.); uzanav@post.bgu.ac.il (A.Y.U.); ofbr@post.bgu.ac.il (O.B.); 2 Nuclear Research Center-Negev, P.O. Box 9001, Beer-Sheva 84190, Israel

**Keywords:** biological composites, interfaces, dynamic modulus, analytical modeling, composite mechanics

## Abstract

Biological composites (biocomposites) possess ultra-thin, irregular-shaped, energy dissipating interfacial regions that grant them crucial mechanical capabilities. Identifying the dynamic (viscoelastic) modulus of these interfacial regions is considered to be the key toward understanding the underlying structure–function relationships in various load-bearing biological materials including mollusk shells, arthropod cuticles, and plant parts. However, due to the submicron dimensions and the confined locations of these interfacial regions within the biocomposite, assessing their mechanical characteristics directly with experiments is nearly impossible. Here, we employ composite-mechanics modeling, analytical formulations, and numerical simulations to establish a theoretical framework that links the interfacial dynamic modulus of a biocomposite to the extrinsic characteristics of a larger-scale biocomposite segment. Accordingly, we introduce a methodology that enables back-calculating (via simple linear scaling) of the interfacial dynamic modulus of biocomposites from their far-field dynamic mechanical analysis. We demonstrate its usage on zigzag-shaped interfaces that are abundant in biocomposites. Our theoretical framework and methodological approach are applicable to the vast range of biocomposites in natural materials; its essence can be directly employed or generally adapted into analogous composite systems, such as architected nanocomposites, biomedical composites, and bioinspired materials.

## 1. Introduction

Load-bearing biocomposites are typically structured as arrays of rigid and predominantly elastic reinforcing elements (e.g., biominerals or crystalline biopolymers), which are connected by a more compliant and energy-dissipating matrix material (e.g., proteins or hemicellulose) through submicron length, compositionally graded, and irregularly-shaped interfacial regions [1,2,3,4,5,6,7,8]. The effective dynamic (viscoelastic) modulus of these interfacial regions provides the biocomposites’ diverse mechanical functions, including adsorbing impacts, detaining cracks, and filtering mechanical signals [9,10,11,12,13,14,15,16,17]. Identifying the interfacial dynamic modulus of biocomposites is a long-standing objective of biomaterial science research [18], it is considered the keystone toward understanding the fundamental structure–function relationships in various biocomposite systems [19,20,21,22,23,24,25].

Nanomechanical testing methods, e.g., nanoindentation and nanoscale dynamic mechanical analysis (DMA), are the benchmark approaches to identifying the mechanical characteristics of the interfacial regions in biocomposites [18,26,27,28,29,30,31,32]. These methods apply local contact loadings to certain locations within the interfacial region, analyze their mechanical response upon static or harmonic forces, and determine the elastic stiffness and viscous damping characteristics of the underlying reinforcement or matrix materials within the interfacial region. These underlying material characteristics link to the mechanical response of the interfacial region as a whole via shear-lag mechanisms, which transfer axial loads between adjacent reinforcements through the tensile-shear loadings of their intermediate matrix material [33,34,35,36]. Recent studies on planar interfacial morphologies (e.g., staggered, triangular, and trapezoid) have derived analytical relationships between the overall dynamic modulus of the interfacial region and those of its underlying reinforcement and matrix materials [37,38,39,40,41]. However, these analytical relationships cannot account for non-planar, irregularly-shaped, or unmarked interfacial morphologies (as commonly present in natural materials), which must be characterized through direct interfacial experiments. Practically, such direct interfacial experiments are nearly impossible due to the small dimensions and the confined locations of the interfacial regions within the biocomposite complex. The interfacial mechanical characteristics must be analytically extracted from far-field experiments on a larger-scale biocomposite segment [42,43,44,45,46]. Even small variations in the interfacial characteristics, i.e., material properties or relative content within the biocomposite, may substantially affect the mechanical response of the biocomposite segment [47,48,49]. Establishing feasible methodologies to approach the interfacial dynamic modulus of biocomposites from far-field experiments is a pending challenge of both biological and synthetic nanomaterial science [50,51,52,53,54].

Here, we employ composite-mechanics modeling, theoretical approximations, and numerical simulations to identify simple analytical relationships between the dynamic modulus (i.e., modulus magnitude and loss coefficient) of an interfacial region within a biocomposite to its larger-scale, enclosing biocomposite segment. With these relationships, we propose an analytical methodology that allows for the back-calculation (linear scaling) of the interfacial dynamic modulus from far-field DMA results on the biocomposite segment. Finally, we demonstrate the usability and adequacy of our methodology via numerical experiments on a class of sutural interfaces that are abundant in natural materials.

## 2. Analytical Relationships for the Interface–Biocomposite Dynamic Moduli

**Figure 1:**
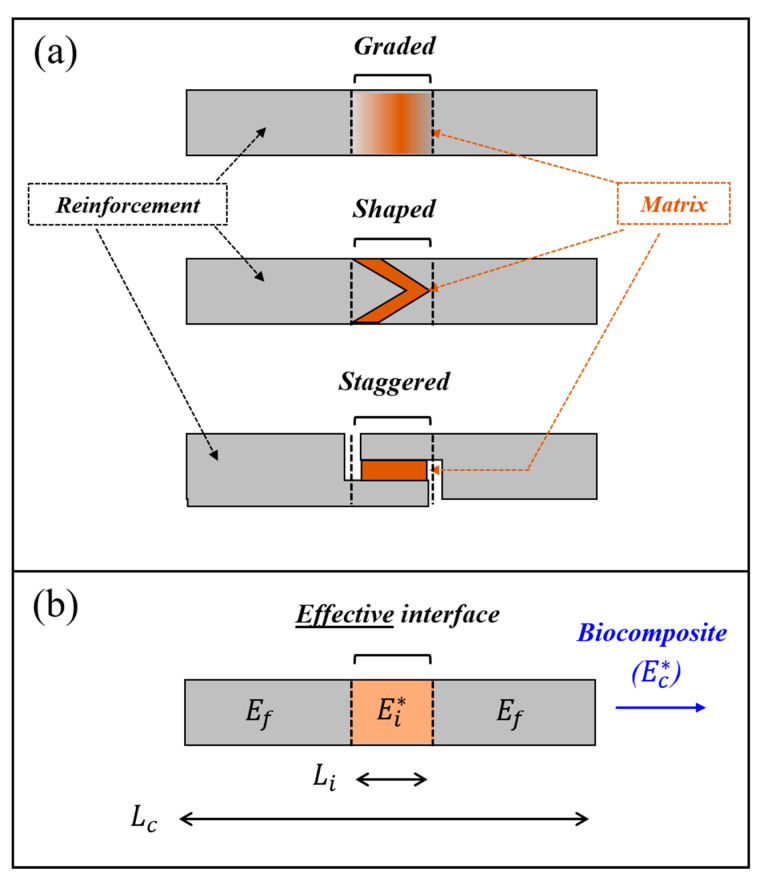
(**a**) Schematic examples of the interfacial regions in biocomposites: graded matrix properties, shaped matrix-reinforcement connection, and staggered matrix-reinforcement connection. (**b**) Mechanical modeling of a biocomposite segment (length $L_{c}$ and dynamic modulus $E_{c}^{*}$), which includes an effective viscoelastic interface (length $L_{i}$ and dynamic modulus $E_{i}^{*}$) between adjacent elastic reinforcements (elastic modulus $E_{f}$).

We considered a biocomposite segment—an isolated specimen from a larger-scale biocomposite complex—that includes adjacent elastic reinforcements connected by an energy-dissipating, viscoelastic matrix in various possible structural forms (Figure 1a). We identified the interfacial region (of length $L_{i}$) within the biocomposite segment (of length $L_{c}$) as the region that is different from the pristine reinforcements in terms of material properties or architectural characteristics. We characterized the elastic behavior of the reinforcements via Young’s modulus $E_{f}$ and the viscoelastic behavior of the whole biocomposite segment via the dynamic (complex) modulus $E_{c}^{*}=E_{c}\cdot e^{j\cdot \delta_{c}}$, where $E_{c}$ is the modulus magnitude of the biocomposite, and $\tan\delta_{c}$ is the loss coefficient of the biocomposite. We characterized the viscoelastic behavior of the interfacial region by an effective dynamic modulus $E_{i}^{*}=E_{i}\cdot e^{j\cdot \delta_{i}}$ that compiles the properties of its underlying reinforcement and matrix materials via the interfacial shear-lag mechanisms ($E_{i}$ and $\tan\delta_{i}$ are the effective modulus magnitude and the effective loss coefficient of the interfacial region, respectively).

To connect the interfacial dynamic modulus ($E_{i}^{*}$) to that of the whole biocomposite segment ($E_{c}^{*}$), we modeled the biocomposite segment as a reinforcement-interface-reinforcement sequence (Figure 1b) and employed Reuss’s model from classical composite mechanics (adapted via the correspondence principle) [49,55], which aptly describes various analogous biocomposite configurations, both at the macromolecular level (e.g., nanofibrils) [44], the nanocomposite level (e.g., nanofibril arrays enriched with biominerals) [3], and the microcomposite level (e.g., lamellar architectures) [56]. Consequentially, we expressed $E_{i}^{*}$ as follows [49,55]:

(1) $$E_{i}^{*}={\left[\frac{1/\left(L_{i}/L_{c}\right)\;}{E_{c}^{*}}-\frac{1/\left(L_{i}/L_{c}\right)-1}{E_{f}}\right]}^{-1}$$

Practically, the biocomposite dynamic modulus ($E_{c}^{*}$) is characterized by small-scale DMA experiments, e.g., atomic force microscopy or dynamic nanoindentation, which yield $E_{c}$ and $\tan\delta_{c}$ from the amplitude ratio and the phase shift between the harmonic stress and strain signals, respectively. Thus, we wished to draw direct analytical connections between these experimental measures ($E_{c}$ and $\tan\delta_{c}$) to the corresponding characteristics of the interfacial region ($E_{i}$ and $\tan\delta_{i}$). By employing standard analytical steps (see Section S1 in Supporting Information) into Equation (1), we obtained the following equivalent equations:

(2) $$E_{i}=E_{c}\cdot \frac{L_{i}}{L_{c}}\cdot \frac{1}{\sqrt{1-2\cdot \left(1-\frac{L_{i}}{L_{c}}\right)\cdot \left(\frac{E_{c}}{E_{f}}\right)\cdot \frac{1}{\sqrt{1+\tan^{2}\delta_{c}}}+{\left(\frac{E_{c}}{E_{f}}\right)}^{2}\cdot {\left(1-\frac{L_{i}}{L_{c}}\right)}^{2}}}$$

(3) $$\tan\delta_{i}=\tan\delta_{c}\cdot \frac{1}{1-\frac{E_{c}}{E_{f}}\cdot \left(1-\frac{L_{i}}{L_{c}}\right)\cdot \sqrt{1+\tan^{2}\delta_{c}}}$$

Next, we considered the typical mechanical characteristics of biocomposites, $E_{c}/E_{f}\leq 1/4$ and $\tan\delta_{c}\leq 1/2$ (which are mostly far below these bounds; see [18] and the references therein), introduced analytical approximations into Equations (2) and (3), and obtained the following relationships (see section S1 in Supporting Information):

(4) $$E_{i}=k_{E}\cdot E_{c};\;k_{E}=\frac{L_{i}/L_{c}}{1-\left(E_{c}/E_{f}\right)\cdot \left(1-L_{i}/L_{c}\right)}$$

(5) $$\tan\delta_{i}=k_{\delta }\cdot \tan\delta_{c};\;k_{\delta }=\frac{1}{1-\left(E_{c}/E_{f}\right)\cdot \left(1-L_{i}/L_{c}\right)}$$

Equations (4) and (5) show that the interface–biocomposite characteristics, $E_{i}–E_{c}$ and $\tan\delta_{i}–\tan\delta_{c}$, link via linear scaling. The scaling factors, $k_{E}$ and $k_{\delta }$, depend only on the biocomposite-to-reinforcement modulus ratio ($E_{c}/E_{f}$) and the relative length of the interface within the biocomposite ($L_{i}/L_{c}$), and exclude the loss coefficient of the biocomposite ($\tan\delta_{c}$). Specifically, when the biocomposite-to-reinforcement modulus ratio is sufficiently small ($E_{c}/E_{f}<1/10$), or when the relative length of the interfacial region is sufficiently large ($1/2<L_{i}/L_{c}$), the denominators of $k_{E}$ and $k_{\delta }$ approaches unity, and Equations (4) and (5) further simplify into $E_{i}\approx L_{i}/L_{c}\cdot E_{c}$ and $\tan\delta_{i}\approx \tan\delta_{c}$. Notably, as $k_{E}$ and $k_{\delta }$ are nondimensional, the $E_{i}–E_{c}$ and $\tan\delta_{i}–\tan\delta_{c}$ relationships in Equations (4) and (5) are independent of the absolute length scale and the mechanical characteristics of the biocomposite. Consequentially, these $E_{i}–E_{c}$ and $\tan\delta_{i}–\tan\delta_{c}$ relationships are generally applicable for the broad dimensional range of biocomposites in natural materials—including macromolecular, nanoscale, and microscale biocomposites—and their diverse mechanical characteristics, i.e., from highly rigid to substantially compliant and from nearly elastic to prominently viscous.

**Figure 2:**
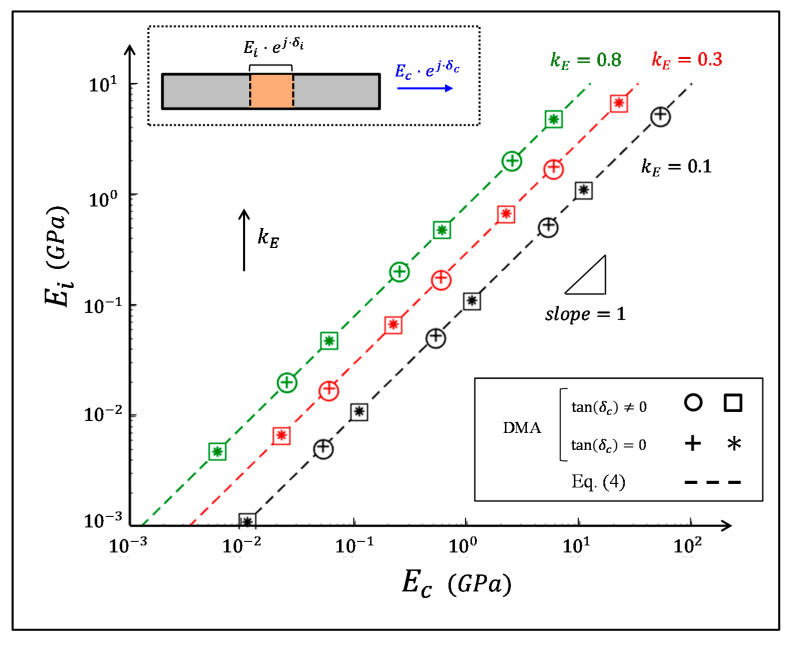
The correspondence between $E_{i}$ and $E_{c}$ for various biocomposite configurations. The dashed lines indicate the theoretical results via Equation (4), and the symbols indicate the corresponding finite-element results (Table S1 in Supporting Information). The colors indicate data sets with the same $k_{E}$ parameter: black, red, and green correspond to $k_{E}=0.1,\;\;0.3$, and 0.8, respectively. The circle and square symbols represent different viscoelastic biocomposite configurations ($\tan\delta_{c}\neq 0$), while the plus and asterisk symbols represent the corresponding biocomposite configurations, but with completely elastic properties ($\tan\delta_{c}=0$).

**Figure 3:**
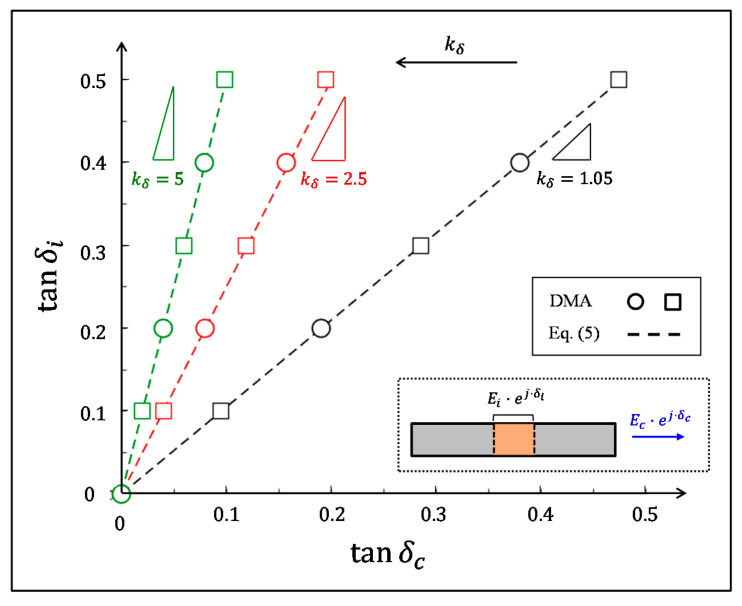
The correspondence between $\tan\delta_{i}$ and $\tan\delta_{c}$ for various biocomposite configurations. The dashed lines indicate the theoretical results via Equation (4), and the symbols indicate the corresponding finite-element results (Table S2 in Supporting Information). The colors indicate data sets with the same $k_{\delta }$ parameter: black, red, and green correspond to $k_{\delta }=1.05,\;2.5$, and 5, respectively. The range of $\tan\delta_{i}$ spans between completely elastic ($\tan\delta_{i}=0$) and predominantly viscoelastic ($\tan\delta_{i}=1/2$) interfaces.

We employed numerical dynamic mechanical analysis via finite element (FE) simulations (Abaqus 6.12, T2D2H elements) to verify the interface–biocomposite relationships in Equations (4) and (5). We analyzed a biocomposite segment (length $L_{c}$) that was comprised of a pair of elastic reinforcements (elastic modulus $E_{f}$) and an intermediate viscoelastic interface (length $L_{i}$ and dynamic modulus $E_{i}^{*}=E_{i}\cdot e^{j\cdot \delta_{m}}$). In each simulation, we applied harmonic strain loadings on the lateral edges of the biocomposite segment, probed the resulting harmonic stresses on these edges, and extracted the modulus magnitude and the loss coefficient of the biocomposite ($E_{c}$ and $\tan\delta_{c}$) from the amplitude ratio and the phase shift between the stress and strain signals. We analyzed the simulation models with a wide range of input parameters that enclosed the typical characteristics of natural materials (see Tables S1 and S2 in the Supporting Information), we plotted their resultant pairs, $E_{i}-E_{c}$ pairs (Figure 2), and $\tan\delta_{i}-\tan\delta_{c}$ pairs (Figure 3). For each simulation model, we calculated the biocomposite-to-reinforcement modulus ratio ($E_{c}/E_{f}$), the relative length of the interfacial region within the biocomposite ($L_{i}/L_{c}$), and the resultant interface–biocomposite scaling factors ($k_{E}$ and $k_{\delta }$, respectively). Then, we plotted the theoretical relationships in Equations (4) and (5) with the $E_{i}-E_{c}$, and $\tan\delta_{i}-\tan\delta_{c}$ pair sets from the numerical simulations (Figure 2 and Figure 3) which showed excellent correspondence for the entire range of input parameters analyzed.

## 3. Assessing the Interfacial Dynamic Modulus from a Far-Field Dynamic Mechanical Analysis

### 3.1. Methodological Approach

We have used the interface–biocomposite relationships in Equations (4) and (5) to propose the following methodological approach to back-calculate the interfacial dynamic modulus from far-field DMA measurements on the biocomposite segment itself.

*Step 1:* Isolate a testing segment (length $L_{c}$) from the biocomposite complex and use microscopy observations to identify its underlying interfacial region (length $L_{i}$).
*Step 2:* Apply DMA testing on a biocomposite segment and quantify its modulus magnitude and loss coefficient ($E_{c}$ and $\tan\delta_{c}$).
*Step 3:* Use nanomechanical testing (or the literature data) to determine the elastic modulus of the reinforcements ($E_{f}$) outside the interfacial region.
*Step 4:* Calculate the interface–biocomposite scaling factors ($k_{E}$ and $k_{\delta }$), and use them to back-calculate the modulus magnitude and the loss coefficient of the interfacial region from the corresponding biocomposite characteristics ($E_{i}=k_{E}\cdot E_{c}$, and $\tan\delta_{i}=k_{\delta }\cdot \tan\delta_{c}$).

Practically, the estimations of $L_{i}$ and $E_{f}$ (Steps 1 and 3 above) typically incorporate certain deviation ranges, which arise from the resolution limitation of the microscopy analysis, and from the inherent variability of the nanomechanical analysis, respectively, such that $L_{i}\to L_{i}\pm \Delta L_{i}$, and $E_{f}\to E_{f}\pm \Delta E_{f}$. Accordingly, the interface–biocomposite scaling factors also include deviation ranges $k_{E}\to k_{E}\pm \Delta k_{E}$, and $k_{\delta }\to k_{\delta }\pm \Delta k_{\delta }$. To identify these deviations, we calculated the first-order differentials of Equations (4) and (5), namely $\Delta k_{E}=dk_{E}/dL_{i}\;\cdot \Delta L_{i}+dk_{E}/dE_{f}\cdot \Delta E_{f}$, and $\Delta k_{\delta }=dk_{\delta }/dL_{i}\cdot \Delta L_{i}+dk_{\delta }/dE_{f}\cdot \Delta E_{f}$, and we expressed them analytically as follows:

(6) $$\Delta k_{E}=k_{E}\cdot \left[\left(1-A\right)\cdot \frac{\Delta L_{i}}{L_{i}}-B\cdot \frac{\Delta E_{f}}{E_{f}}\right]$$

(7) $$\Delta k_{\delta }=k_{\delta }\cdot \left[-A\cdot \frac{\Delta L_{i}}{L_{i}}-B\cdot \frac{\Delta E_{f}}{E_{f}}\right]$$

where

(8) $$A=\frac{L_{i}}{L_{c}}\cdot \frac{E_{c}}{E_{f}}\cdot \frac{1}{C}\;,\;\;\;\;\;B=\frac{E_{c}}{E_{f}}\cdot \left[1-\frac{L_{i}}{L_{c}}\right]\cdot \frac{1}{C}\;,\;\;\;\;\;C=1-\left(\frac{E_{c}}{E_{f}}\right)\cdot \left(1-\frac{L_{i}}{L_{c}}\right)$$

Notably, when the interfacial region occupies large portions of the biocomposite segment ($L_{i}/L_{c}\to 1$), the coefficients B≪A, and the $k_{E}$ and $k_{\delta }$ deviations reduce into $\Delta k_{E}\approx k_{E}\cdot \left(1-A\right)\cdot \Delta L_{i}/L_{i}$ and $\Delta k_{\delta }\approx -k_{\delta }\cdot A\cdot \Delta L_{i}/L_{i}$; both $k_{E}$ and $k_{\delta }$ are insensitive to $E_{f}$ deviations. Moreover, when the biocomposite-to-reinforcement modulus ratio is sufficiently small ($E_{c}/E_{f}<1/10$), the coefficients A,B≪1, and the $k_{E}$ and $k_{\delta }$ deviations, further simplify into $\Delta k_{E}\approx k_{E}\cdot \Delta L_{i}/L_{i}$ and $\Delta k_{\delta }\ll k_{\delta }$; the $k_{E}$ deviations are proportional to the $L_{i}$ deviations, and the $k_{\delta }$ deviations are approximately negligible.

Next, we demonstrate our approach on biocomposites with zigzag-shaped sutural interfaces and illustrate the effect of their zigzag angles on the modulus magnitude and the loss coefficient of these interfacial regions.

### 3.2. Example: Sutural Interfaces

**Figure 4:**
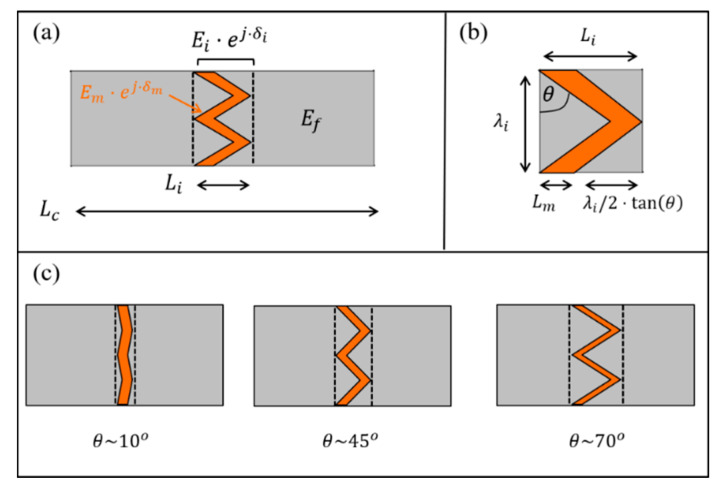
Schematic descriptions of zigzag-shaped sutural interfaces in biocomposites and their geometrical parameters. (**a**) A biocomposite segment with an underlying sutural interface. (**b**) An isolated interfacial region (one period) from the biocomposite. (**c**) Schematic examples of sutural interfaces with the same matrix length ($L_{m}$), but different zigzag angles (θ=10°, 45°, and ~70°).

**Figure 5:**
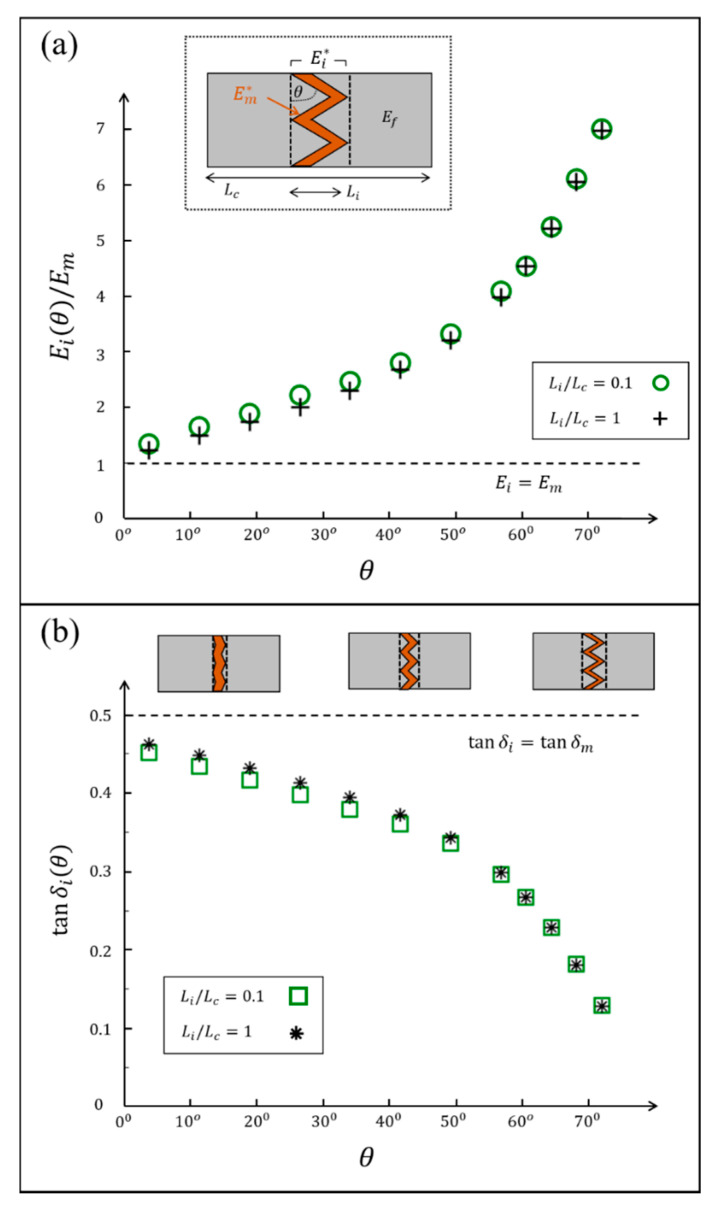
Simulation results for the interfacial dynamic modulus of biocomposites with zigzag-shaped sutural interfaces. (**a**) the modulus magnitude and (**b**) the loss coefficient of the interfacial region for different zigzag angles (θ), achieved by back-calculations from far-field DMA testing on the biocomposite ($L_{i}/L_{c}=0.1$), and by direct DMA testing on the interfacial region ($L_{i}/L_{c}=1$).

Sutural interfaces are abundant in natural materials, such as dermal armors [57,58], bird beaks [59], and seed coats [60], and they serve as locally flexible and energy-dissipating regions within a much harder and fragile bulk material. These sutural interfaces commonly view periodic zigzag geometries (period length $\lambda_{i}$, zigzag angle θ), which connect adjacent elastic reinforcements (elastic modulus $E_{f}$) via a viscoelastic matrix layer (length $L_{m}$, dynamic modulus $E_{m}^{*}=E_{m}\cdot e^{j\cdot \delta_{m}}$), and yield an interfacial region of length $L_{i}=L_{m}+\lambda_{i}/2\cdot \tan\theta$ (Figure 4). The zigzag angle of these sutural interfaces plays a major role in the various mechanical functions of the sutural interfaces, e.g., its elastic stiffness, failure strength, dumping capability, and impact resistance [11,36,61,62]. Here, we use numerical simulations to demonstrate the effect of the zigzag angle of the sutural interface on its effective dynamic modulus [41]. We focus on sutural interfaces with substantial matrix portions ($L_{m}/\lambda_{i}=1/4$) and small-to-moderate zigzag angles (θ=0→75°), which complement the framework of corresponding analytical models and extract the effective modulus magnitude and loss coefficient of these interfacial regions from a far-field DMA analysis of a biocomposite segment via the above methodological approach (Section 3.1). We used plane stress FE simulations (Abaqus 6.12, CPS4 elements) with a substantially viscoelastic matrix (loss coefficient $\tan\delta_{m}=1/2$), and much stiffer, elastic reinforcements ($E_{f}/E_{m}=10$). For simplicity, we considered the matrix and reinforcements to be nearly incompressible ($E_{f}/G_{f}=E_{m}^{*}/G_{m}^{*}=2.5$). For each zigzag angle analyzed (θ), we realized the geometry of the sutural interface ($L_{m}/\lambda_{i}=1/4$, and $L_{i}/L_{m}=1+2\cdot \tan\theta$), and set the remaining length of the reinforcements to ensure that the overall interface-to-composite length ratio was kept constant ($L_{i}/L_{c}=0.1$). For each case, we applied harmonic strain loadings on the lateral edges of the biocomposite (upper and lower edges were free), measured the resulting harmonic stresses on these edges, and extracted the modulus magnitude and the loss coefficient of the biocomposite ($E_{c}\left(\theta \right)/E_{m}$ and $\tan\delta_{c}\left(\theta \right)$) from the amplitude ratio and the phase shift between the stress and strain signals. Then, we calculated the scaling factors $k_{E}$ and $k_{\delta }$ factors, and back-calculated the modulus magnitude and the loss coefficient of the sutural interface, $E_{i}\left(\theta \right)/E_{m}$ and $\tan\delta_{i}\left(\theta \right)$), via Equations (4) and (5), respectively (Figure 5a,b, Table S3 in Supporting Information). To verify our results, we performed a complementary analysis on the isolated sutural interfaces (i.e., $L_{i}/L_{c}=1$) that directly yielded $E_{i}\left(\theta \right)/E_{m}$ and $\tan\delta_{i}\left(\theta \right)$ (i.e., without back-calculations); evidently, our estimations from the far-field DMA analysis on the biocomposite segments are in good agreement with the direct analysis on the isolated sutural interfaces for the entire θ range analyzed. Our results show that the zigzag angle strongly affects the modulus magnitude and the loss coefficient of the sutural interface (Figure 5a,b), and are in line with the theoretical trends of recent analytical studies [41]. As the zigzag angle approaches zero, the interfacial region is almost flat and mostly comprises the matrix layer ($L_{i}/L_{m}\sim 1$), and its modulus magnitude and loss coefficient approach that of the pristine matrix material $E_{i}\left(\theta \to 0\right)\sim E_{m}$ and $\tan\delta_{i}\left(\theta \to 0\right)\sim \tan\delta_{m}$. Conversely, as the zigzag angle increases, the interfacial region occupies greater portions of the stiffer elastic reinforcements (in addition to the matrix layer), which results in a progressive increase in the modulus magnitude of the interfacial region $E_{i}\left(\theta \right)$ and a complementary decrease in its loss coefficient $\tan\delta_{i}\left(\theta \right)$. Thus, natural sutural interfaces with similar matrix and reinforcement characteristics can achieve different biomechanical functions merely through morphological adaptations of their zigzag shapes. Mild zigzag shapes will generate interfacial regions with greater flexibility and higher energy-dissipation capabilities, whereas acute zigzag shapes will generate interfacial regions with greater robustness and higher energy-storing capabilities.

## 4. Conclusions

The interfacial dynamic modulus of biocomposites dominates the structure–function relationships in various organisms, including vertebrates (e.g., mammals, birds, and fishes), invertebrates (e.g., insects, arachnids, and mollusks), and plants. Nevertheless, due to the small dimensions and the confined locations of these interfacial regions, measuring their direct mechanical characterizations is nearly impossible. In this study, we established compact analytical formulae that link the modulus magnitude and the loss coefficient of the interfacial region to those of its enclosing, large-scale, biocomposite segment. We used these formulae to propose an analytical and experimental methodology that yielded the interfacial characteristics via back-calculations, from a feasible, far-field DMA analysis of the biocomposite itself and demonstrated it on zigzag-shaped sutural interfaces. From a broader perspective, our approach can also be used to analyze the interfacial characteristics of advanced engineering materials, such as bioinspired composites, nanocomposites, and electromechanical devices [63,64,65,66].

## Data Availability

Data is contained within the article or supplementary material.

## Supplementary Materials

The following are available online at https://www.mdpi.com/article/10.3390/ma14123428/s1, Table S1: Summary of DMA simulations results, shown in Figure 2 in the main text; Table S2: Summary of DMA simulations results, shown in Figure 3 in the main text; Table S3: Summary of the DMA simulation results for the zigzag-shaped interfaces and the corresponding back-calculations of $E_{i}$ and $\tan\delta_{i}$ via Equations (4) and (5).
