# Supplementary material for: Assessing the Interfacial Dynamic Modulus of Biological Composites
Source: Materials (Basel). 2021 Jun 21;14(12):3428. doi: 10.3390/ma14123428 (PMC8234923; doi:10.3390/ma14123428)
Supplement: Supplementary file 1 [file materials-14-03428-s001.zip › materials-1236756-supplementary.pdf]

materials

MDPI

Supporting Materials

Assessing the Interfacial Dynamic Modulus of Biological Composites

Yaniv Shelef [1,†], Avihai Yosef Uzan [1,†], Ofer Braunshtein [1,2] and Benny Bar-On [1,*]

[1] Department of Mechanical Engineering, Ben-Gurion University of the Negev, Beer Sheva 84105, Israel; shelefy@post.bgu.ac.il (Y.S.); uzanav@post.bgu.ac.il (A.Y.U.); ofbr@post.bgu.ac.il (O.B.)
[2] Nuclear Research Center-Negev, P.O. Box 9001, Beer-Sheva 84190, Israel
* Correspondence: bbo@bgu.ac.il
† Authors with equal contributions.

S1. Derivation of the analytical formulae

We consider a biocomposite segment of length L_c and a dynamic modulus $E_c^* = E_c \cdot e^{i \cdot \delta_c}$, which includes a pair of elastic reinforcements of elastic modulus E_f —connected by a viscoelastic interface of length L_i and a dynamic modulus $E_i^* = E_i \cdot e^{j \cdot \delta_i}$ (Figure 1b, main text). Using the inverse rule-of-mixtures, E_c^* links to E_f and E_i^* via the relative length of the interface in the biocomposite segment (L_i/L_c):

$$\frac{1}{E_c^*} = \frac{1 - L_i/L_c}{E_f} + \frac{L_i/L_c}{E_i^*} \tag{S1}$$

By rearranging Equation (S1), we obtain:

$$E_i^* \cdot \left[E_f - E_c^* \cdot (1 - L_i/L_c)\right] = E_c^* \cdot E_f \cdot L_i/L_c \tag{S2}$$

Next, we express the dynamic moduli of the interfacial region and the composite segment via their substitute real and imaginary parts, $E_i^* = E_i' + j \cdot E_i''$ and $E_c^* = E_c' + j \cdot E_c''$, substitute them into Equation (S2), and solve the real and imaginary parts, respectively:

$$E_i' \cdot E_f - E_i' \cdot E_c' \cdot (1 - L_i/L_c) + E_i'' \cdot E_c'' \cdot (1 - L_i/L_c) = E_c' \cdot E_f \cdot L_i/L_c \tag{S3}$$

$$E_c'' \cdot E_i' \cdot (1 - L_i/L_c) + E_i'' \cdot E_f - E_i'' \cdot E_c' \cdot (1 - L_i/L_c) = E_c'' \cdot E_f \cdot L_i/L_c \tag{S4}$$

Then, we solve Equations (S3a) and (S3b) for E_i' and E_i'' and obtain:

$$E_i' = \frac{E_f \cdot L_i/L_c \cdot \left[\left({E_c'}^2 + {E_c''}^2\right) \cdot (L_i/L_c - 1) + E_c' \cdot E_f\right] \cdot}{{E_c'}^2 \cdot (L_i/L_c - 1)^2 + 2 \cdot E_c' \cdot E_f \cdot (L_i/L_c - 1) + {E_c''}^2 \cdot (L_i/L_c - 1)^2 + E_f^2} \tag{S5}$$

$$E_i'' = \frac{E_c'' \cdot E_f^2 \cdot L_i/L_c}{{E_c'}^2 \cdot (L_i/L_c - 1)^2 + 2 \cdot E_c' \cdot E_f \cdot (L_i/L_c - 1) + {E_c''}^2 \cdot (L_i/L_c - 1)^2 + E_f^2} \tag{S6}$$

Next, express the modulus magnitude of the interfacial region via $E_i = \sqrt{{E_i'}^2 + {E_i''}^2}$, use the relationships $E_c = \sqrt{{E_c'}^2 + {E_c''}^2}$ and $E_c' = E_c \cdot / \sqrt{1 + \tan^2 \delta_c}$, and rearrange to obtain Equation (2) at the main text:

$$E_i = E_c \cdot \frac{L_i}{L_c} \cdot \frac{1}{\sqrt{1 - 2 \cdot \left(1 - \frac{L_i}{L_c}\right) \cdot \left(\frac{E_c}{E_f}\right) \cdot \frac{1}{\sqrt{1 + \tan^2 \delta_c}} + \left(\frac{E_c}{E_f}\right)^2 \cdot \left(1 - \frac{L_i}{L_c}\right)^2}} \tag{S7}$$

Similarly, we express the loss coefficient of the interfacial region via $E_i = E_i''/E_i'$, use again the relationship $E_c' = E_c \cdot / \sqrt{1 + \tan^2 \delta_c}$, and rearrange to obtain Equation (3) at the main text:

$$\tan\delta_i = \tan\delta_c \cdot \frac{1}{1 - \frac{E_c}{E_f}\cdot\left(1 - \frac{L_i}{L_c}\right)\cdot\sqrt{1+\tan^2\delta_c}} \tag{S8}$$

Next, we consider $\tan\delta_c \le 1/2$, for which $\sqrt{1+\tan^2\delta_c} \approx 1 + \frac{1}{2}\cdot\tan^2\delta_c$ and $\frac{1}{\sqrt{1+\tan^2\delta_c}} \approx 1 - \frac{1}{2}\cdot\tan^2\delta_c$, and introduce these approximations into Equations (S7) and (S8):

$$E_i = E_c \cdot \frac{L_i}{L_c}\cdot \frac{1}{\sqrt{\left[1 - \frac{E_c}{E_f}\cdot\left(1 - \frac{L_i}{L_c}\right)\right]^2 + \left(1 - \frac{L_i}{L_c}\right)\cdot\left(\frac{E_c}{E_f}\right)\cdot\tan^2\delta_c}} \tag{S9}$$

$$\tan\delta_i = \tan\delta_c \cdot \frac{1}{\left[1 - \frac{E_c}{E_f}\cdot\left(1 - \frac{L_i}{L_c}\right)\right] - \frac{1}{2}\cdot\frac{E_c}{E_f}\cdot\left(1 - \frac{L_i}{L_c}\right)\cdot\tan^2\delta_c} \tag{S10}$$

Rearranging:

$$E_i = E_c \cdot \frac{L_i}{L_c}\cdot\frac{1}{1 - \frac{E_c}{E_f}\cdot\left(1 - \frac{L_i}{L_c}\right)}\cdot\frac{1}{\sqrt{1 + \frac{\left(1 - \frac{L_i}{L_c}\right)\cdot\left(\frac{E_c}{E_f}\right)\cdot\tan^2\delta_c}{\left[1 - \frac{E_c}{E_f}\cdot\left(1 - \frac{L_i}{L_c}\right)\right]^2}}} \tag{S11}$$

$$\tan\delta_i = \tan\delta_c \cdot \frac{1}{1 - \frac{E_c}{E_f}\cdot\left(1 - \frac{L_i}{L_c}\right)\cdot}\cdot\frac{1}{1 - \frac{\frac{1}{2}\cdot\frac{E_c}{E_f}\cdot\left(1 - \frac{L_i}{L_c}\right)\cdot\tan^2\delta_c}{1 - \frac{E_c}{E_f}\cdot\left(1 - \frac{L_i}{L_c}\right)}} \tag{S12}$$

We consider $0 < \frac{L_i}{L_c}, \frac{E_c}{E_f} \le \frac{1}{4}$ and $\tan\delta_c \le \frac{1}{2}$, use Taylor's expansions for the denominators of the right-hand terms in Equations (S11) and (S12), and identify that:

$$\sqrt{1 + \frac{\left(1 - \frac{L_i}{L_c}\right)\cdot\left(\frac{E_c}{E_f}\right)\cdot\tan^2\delta_c}{\left[1 - \frac{E_c}{E_f}\cdot\left(1 - \frac{L_i}{L_c}\right)\right]^2}} \le \sqrt{1 + \frac{\left(\frac{E_c}{E_f}\right)\cdot\tan^2\delta_c}{\left(1 - \frac{E_c}{E_f}\right)^2}} \le \sqrt{1 + \frac{1}{9}} \sim 1 \tag{S13}$$

$$1 - \frac{\frac{1}{2}\cdot\frac{E_c}{E_f}\cdot\left(1 - \frac{L_i}{L_c}\right)\cdot\tan^2\delta_c}{1 - \frac{E_c}{E_f}\cdot\left(1 - \frac{L_i}{L_c}\right)} \le 1 - \frac{\frac{1}{2}\cdot\frac{E_c}{E_f}\cdot\tan^2\delta_c}{1 - \frac{E_c}{E_f}\cdot} \le 1 - \frac{1}{28} \sim 1 \tag{S14}$$

Consequentially, Equations (S11) and (S12) reduce to Equations (3) and (4) at the main text:

$$E_i = E_c \cdot \frac{L_i}{L_c}\cdot\frac{1}{1 - \frac{E_c}{E_f}\cdot\left(1 - \frac{L_i}{L_c}\right)} \tag{S15}$$

$$\tan\delta_i = \tan\delta_c \cdot \frac{1}{1 - \frac{E_c}{E_f}\cdot\left(1 - \frac{L_i}{L_c}\right)} \tag{S16}$$

Table S1. Summary of DMA simulations results, shown in Figure 2 in the main text.

Input DMA-FE				*Output DMA-FE*		k_E $(Eq. 4)$	*Symbol*
E_f (GPa)	E_i/E_f	L_i/L_c	$\tan\delta_i$	$E_c(GPa)$	$\tan\delta_c$		
0.1	0.053	0.05	0	0.0526	0	0.1	○
1	0.053	0.05	0	0. 526	0	0.1	○
10	0.053	0.05	0	5.26	0	0.1	○
100	0.053	0.05	0	52.6	0	0.1	○
0.1	0.011	0.09	0	0.01	0	0.1	□
1	0.011	0.09	0	0.10	0	0.1	□
10	0.011	0.09	0	1.01	0	0.1	□
100	0.011	0.09	0	10.1	0	0.1	□
0.1	0.053	0.05	0.5	0.0526	0.24	0.1	+
1	0.053	0.05	0.5	0. 526	0.24	0.1	+
10	0.053	0.05	0.5	5.26	0.24	0.1	+
100	0.053	0.05	0.5	52.6	0.24	0.1	+
0.1	0.011	0.09	0.5	0.01	0.45	0.1	*
1	0.011	0.09	0.5	0.10	0.45	0.1	*
10	0.011	0.09	0.5	1.01	0.45	0.1	*
100	0.011	0.09	0.5	10.1	0.45	0.1	*
0.1	0.177	0.15	0	0.0588	0	0.3	○
1	0.177	0.15	0	0.588	0	0.3	○
10	0.177	0.15	0	5.88	0	0.3	○
100	0.177	0.15	0	58.8	0	0.3	○
0.1	0.067	0.25	0	0.0222	0	0.3	□
1	0.067	0.25	0	0.222	0	0.3	□
10	0.067	0.25	0	2.22	0	0.3	□
100	0.067	0.25	0	22.2	0	0.3	□
0.1	0.177	0.15	0.5	0.0588	0.24	0.3	+
1	0.177	0.15	0.5	0.588	0.24	0.3	+
10	0.177	0.15	0.5	5.88	0.24	0.3	+
100	0.177	0.15	0.5	58.8	0.24	0.3	+
0.1	0.067	0.25	0.5	0.0222	0.41	0.3	*
1	0.067	0.25	0.5	0.222	0.41	0.3	*
10	0.067	0.25	0.5	2.22	0.41	0.3	*
100	0.067	0.25	0.5	22.2	0.41	0.3	*
0.1	0.199	0.75	0	0.025	0	0.8	○
1	0.199	0.75	0	0.25	0	0.8	○
10	0.199	0.75	0	2.5	0	0.8	○
100	0.199	0.75	0	25	0	0.8	○
0.1	0.047	0.79	0	0.0059	0	0.8	□
1	0.047	0.79	0	0.059	0	0.8	□
10	0.047	0.79	0	0.59	0	0.8	□
100	0.047	0.79	0	5.9	0	0.8	□
0.1	0.067	0.75	0.5	0.025	0.47	0.8	+
1	0.067	0.75	0.5	0.25	0.47	0.8	+
10	0.067	0.75	0.5	2.5	0.47	0.8	+
100	0.067	0.75	0.5	25	0.47	0.8	+
0.1	0.199	0.79	0.5	0.0059	0.49	0.8	*
1	0.199	0.79	0.5	0.059	0.49	0.8	*

10	0.199	0.79	0.5	0.59	0.49	0.8	*
100	0.199	0.79	0.5	5.9	0.49	0.8	*

Table S2. Summary of DMA simulations results, shown in Figure 3 in the main text.

Input DMA-FE				*Output DMA-FE*		k_δ $(Eq. 5)$	*Symbol*
E_f (GPa)	E_i/E_f	L_i/L_c	$\tan\delta_i$	$E_c(GPa)$	$\tan\delta_c$		
10	0.05	0.5	0	0.95	0	1.05	○
10	0.05	0.5	0.2	0.95	0.190	1.05	○
10	0.05	0.5	0.4	0.95	0.379	1.05	○
10	0.015	0.75	0.1	1.9	0.095	1.05	□
10	0.015	0.75	0.3	1.9	0.285	1.05	□
10	0.015	0.75	0.5	1.9	0.473	1.05	□
10	0.079	0.05	0	6.66	0	2.5	○
10	0.078	0.05	0.2	6.66	0.079	2.5	○
10	0.076	0.05	0.4	6.66	0.157	2.5	○
10	0.167	0.1	0.1	6.32	0.040	2.5	□
10	0.165	0.1	0.3	6.32	0.119	2.5	□
10	0.156	0.1	0.5	6.32	0.194	2.5	□
10	0.04	0.01	0	8.1	0	5	○
10	0.04	0.01	0.2	8.1	0.039	5	○
10	0.04	0.01	0.4	8.1	0.079	5	○
10	0.21	0.05	0.1	8.4	0.020	5	□
10	0.20	0.05	0.3	8.4	0.059	5	□
10	0.19	0.05	0.5	8.4	0.098	5	□

Table S3. Summary of the DMA simulation results for the zigzag-shaped interfaces and the corresponding back-calculations of E_i and $\tan\delta_i$ via Equations (4) and (5), where $E_m = 1$, $E_f/E_m = 10$, and $\tan\delta_m = 1/2$

Input DMA-FE			*Output DMA-FE*			*Back-Calculations*		
θ [°]	L_i/L_m	L_i/L_c	E_c/E_m	$\tan\delta_c$	k_E $(Eq. 4)$	E_i/E_m	k_δ $(Eq. 5)$	$\tan\delta_i$
3.8	1.13	0.1	6.1	0.202	0.22	1.34	2.24	0.453
		1	1.22	0.462	1	1.22	1	0.462
11.4	1.40	0.1	6.6	0.175	0.25	1.65	2.48	0.434
		1	1.49	0.448	1	1.49	1	0.448
18.9	1.68	0.1	7.0	0.153	0.27	1.89	2.72	0.416
		1	1.74	0.432	1	1.74	1	0.432
26.5	2.00	0.1	7.4	0.134	0.30	2.22	2.96	0.397
		1	2.00	0.413	1	2.00	1	0.413
34.1	2.35	0.1	7.7	0.118	0.32	2.46	3.22	0.380
		1	2.30	0.394	1	2.30	1	0.394
41.7	2.78	0.1	8.0	0.102	0.35	2.80	3.53	0.360
		1	2.68	0.372	1	2.68	1	0.372
49.3	3.33	0.1	8.3	0.085	0.40	3.32	3.98	0.338
		1	3.20	0.343	1	3.20	1	0.343
56.8	4.06	0.1	8.7	0.064	0.47	4.09	4.66	0.298
		1	3.98	0.299	1	3.98	1	0.299
60.6	4.55	0.1	8.9	0.052	0.51	4.54	5.14	0.267
		1	3.98	0.299	1	3.98	1	0.299
64.4	5.17	0.1	9.2	0.040	0.57	5.24	5.75	0.230
		1	5.22	0.228	1	5.22	1	0.228
68.2	6	0.1	9.4	0.028	0.65	6.11	6.47	0.181
		1	6.05	0.180	1	6.05	1	0.180
72.0	7.16	0.1	9.6	0.018	0.73	7.00	7.29	0.131
		1	6.97	0.128	1	6.97	1	0.128
